# Quick Automatic Synthesis of Solvent-Free 16α-[^18^F] Fluoroestradiol: Comparison of Kryptofix 222 and Tetrabutylammonium Bicarbonate

**DOI:** 10.3389/fonc.2020.577979

**Published:** 2020-09-25

**Authors:** Xiao Jiang, Yingchun Li, Xiaoxiong Wang, Taipeng Shen, Xiuli Li, Yutang Yao, Ge Zhang, Ying Kou, Jiaqi Shen, Zhifu Luo, Zhuzhong Cheng

**Affiliations:** ^1^PET/CT Center, Sichuan Cancer Center, School of Medicine, Sichuan Cancer Hospital & Institute, University of Electronic Science and Technology of China, Chengdu, China; ^2^Radiation Oncology Key Laboratory of Sichuan Province, Sichuan Cancer Center, School of Medicine, Sichuan Cancer Hospital & Institute, University of Electronic Science and Technology of China, Chengdu, China; ^3^Institute of Isotope, China Institute of Atomic Energy, Beijing, China; ^4^Department of Nuclear Medicine & Radiotherapy, Air Force Hospital of Western Theater Command, Chengdu, China

**Keywords:** 16α-[^18^F]fluoroestradiol, estrogen receptor, automatic synthesis, phase transfer catalyst, breast cancer

## Abstract

Estrogen receptor (ER) expression level of human breast cancer often reflects the stage of disease and is usually monitored by immunohistochemical staining *in vitro*. The preferable non-invasive and real-time diagnosis *in vivo* is more accessible by PET scan using 16α-[^18^F]FES. The objective of this study was to develop a quick automatic method for synthesis of solvent-free 16α-[^18^F]FES using a CFN-MPS-200 synthesis system and compare the catalytic efficiency of two phase transfer catalysts, Kryptofix 222/K_2_CO_3_ (K222/K_2_CO_3_) and tetrabutylammonium hydrogen carbonate (TBA·HCO_3_). In this method, phase transfer catalysts K222/K_2_CO_3_ and TBA·HCO_3_ were used, respectively. The intermediate products were both hydrolyzed with hydrochloric acid and neutralized with sodium bicarbonate. The crude product was purified with semi-preparative HPLC, and the solvent was removed by rotary evaporation. The effects of radiofluorination temperature and time on the synthesis were also investigated. Radiochemical purity of solvent-free product was above 99% and the decay-corrected radiochemical yield of 16α-[^18^F]FES was obtained in 48.7 ± 0.95% (catalyzed by K222/K_2_CO_3_, *n* = 4) and 46.7 ± 0.77% (catalyzed by TBA·HCO_3_, *n* = 4, respectively). The solvent-free 16α-[^18^F]FES was studied in clinically diagnosed breast cancer patients, and FES-PET results were compared with pathology diagnosis results to validate the diagnosis value of 16α-[^18^F]FES. The new method was more reliable, efficient, and time-saving. There was no significant difference in catalytic activity between K222/K_2_CO_3_ and TBA·HCO_3_.

## Introduction

Estrogen receptor (ER) expression level in breast cancer is known as a meaningful prognostic indicator. ER-positive breast cancer is considered less aggressive and more sensitive to appropriate hormone therapies (1, 2). The traditional method for monitoring ER expression level is immunohistochemical staining with tumor biopsy samples *in vitro*, which is an invasive technique and cannot reflect the real-time ER expression level of primary and metastatic breast cancer or other tissues. An ideal non-invasive method monitoring real-time ER expression in the whole body is preferable in clinical practice. 16α-[^18^F]FES, an ER imaging biomarker, shows high binding affinity to ER. The utility of 16α-[^18^F]FES, especially in conjunction with [^18^F]fluorodeoxyglucose, is considered of important diagnostic value.

It is a challenge for the routine synthesis of 16α-[^18^F]FES to meet clinical requirements. The first synthesis of 16α-[^18^F]FES was reported by Kiesewetter (3). In recent years, the synthesis of 16α-[^18^F]FES through different methods has been reported by several studies. However, most of these studies required more than 60 min with a low yield and mixed radiochemical impurities. Besides, whether K222/K_2_CO_3_ or TBA·HCO_3_ was the appropriate phase transfer catalyst for automated synthesis of 16α-[^18^F]FES has not been investigated in these studies. Clinical applications of 16α-[^18^F]FES as an important tool to non-invasively measure ER expression will depend on its ideal radiochemical purity and steady routine production with a high yield.

In this study, we developed an automated, reliable, and time-saving method for the synthesis of solvent-free 16α-[^18^F]FES using a rotatory evaporator-equipped module with a commercially available precursor. We also investigated whether K222/K_2_CO_3_ or TBA·HCO_3_ was the appropriate phase transfer catalyst and checked the quality of the radiopharmaceutical catalyzed by K222/K_2_CO_3_ or TBA·HCO_3_ for potential clinical use. The solvent-free 16α-[^18^F]FES was studied in clinically diagnosed breast cancer patients, and FES-PET results were compared with pathology diagnosis results to validate the diagnosis value of 16α-[^18^F]FES.

## Materials and Methods

### General

Reagents and solvents were purchased from Aldrich (Sigma-Aldrich, MO, USA) without further purification. The precursor, 3-methoxymethyl-16β,17β-epiestriol-*O*-cylic sulfone (MMSE), Kryptofix 222 (K222), and 16α-[^19^F]FES reference standard were obtained from ABX (ABX advanced biochemical compounds GmbH, Radeberg, Germany). Cartridges were purchased from Waters (Waters, MA, USA). Before use, QMA cartridge was flushed with 10 ml of potassium carbonate followed by 40 ml of water.

For automatic synthesis, CFN-MPS-200 module (Sumitomo Heavy Industries, Tokyo, Japan) was used and modified. Synthesis software was programmed in the Cupid system (Sumitomo Heavy Industries, Tokyo, Japan). Radioactivity was counted in a dose calibrator (Capintec, NJ, USA). The semi-preparative HPLC (PU-2086 Plus, JASCO, Tokyo, Japan) equipped with a semi-preparative C18 column (YMC, YMC-Pack ODS-AM, 10 × 250 mm, 5 μm), UV/Vis detector (fixed wavelength 280 nm, UV-2075 Plus, JASCO, Tokyo, Japan), and a radiation detector was used to purify the crude product at a flow rate of 4 ml/min, using 30% acetonitrile:30% ethanol:40% water (*v:v:v*) as the mobile phase. Analytical HPLC (Shimadzu LC-15, Suzhou, China) was used for the quality control of the final product, equipped with a UV/Vis detector preset to 280 nm, an analytical C18 column (Shimadzu WondaSil, 4.6 × 250 mm, 5 μm), and a radioactive detector (Eckert & Ziegler, GA, USA). The column flow rate was 1 ml/min and was kept at approximately room temperature. The samples were eluted with a mobile phase of 30% acetonitrile:70% water (*v:v*).

### Automated Synthesis of 16α-[^18^F]FES With Kryptofix 222

Based on ^18^O(p, n)^18^F nuclear reaction, 1.8 ml of target water containing [^18^O]H_2_O was irradiated with protons in a Sumitomo HM-10 cyclotron system (Sumitomo Heavy Industries, Tokyo, Japan) to produce [^18^F]fluoride. The energy of incident protons was 10 MeV, and the beam current was about 60 mA. After the irradiation, [^18^F]fluoride was transferred to the collection tube and then passed through a Sep-Pak light QMA cartridge. The trapped [^18^F]fluoride was eluted with 0.9 ml of a mixed aqueous K222/K_2_CO_3_ (22 mg of K222 in 0.7 ml of acetonitrile and 5.8 mg of potassium carbonate in 0.2 ml of ultrapure water) and then the mixed solution was dried up. To make sure that the fluorination reaction was in an anhydrous condition, the residue was further dried by azeotropic distillation with another 0.5 ml of anhydrous acetonitrile. Two milligrams of MMSE in 1 ml of anhydrous acetonitrile was added to the completely dried reactor, and the mixture was heated at 115°C for 15 min. Then, the intermediate product was subsequently hydrolyzed by 1.5 ml of 2 M HCl at 120°C for 3.5 min. After cooling to room temperature, 1.5 ml of 5.6% NaHCO_3_ solution was added to neutralize the reaction mixture. For purification, the crude product was injected into semi-preparative HPLC, and the purification took about 9 min. The purified product was collected in the rotary evaporator to remove solvents and then passed through a sterile 0.22-μm filter with 10 ml of saline containing 1 ml of ethanol.

The analytical HPLC system was adapted to measure the radiochemical and chemical purity of 16α-[^18^F]FES at a flow rate of 1 ml/min using 30% acetonitrile in water by comparing the HPLC spectrum of the 16α-[^19^F]FES reference standard in the same HPLC conditions. For stability test, 16α-[^18^F]FES in the final formula was tested 8 h after the synthesis using HPLC.

To discover how fluorination temperatures and times influence the yield, [^18^F]fluorination was carried out for 5, 10, and 15 min at 105, 115, 125, and 135°C. The overall radiochemical yield was determined by purifying the product by semi-HPLC.

### Automated Synthesis of 16α-[^18^F]FES With Tetrabutylammonium

To compare chemical purity and radiochemical yield, 16α-[^18^F]FES synthesis processes catalyzed by tetrabutylammonium bicarbonate (TBA·HCO_3_) were the same compared with those catalyzed by K222 except that [^18^F]fluoride trapped in the QMA cartridge was eluted by 500 μl of 0.075 M TBA·HCO_3_ dissolved in ethanol, and fluorination was achieved at 135°C for 10 min.

Residual TBA content in the final product was determined by TLC. Silica plates were spotted with 5 μl of the final product, dried, and visualized with iodine. Brown spots will appear if TBA exists in the final product.

### Patients and PET/CT Procedure

Nine patients (mean age ± SD, 50.2 ± 7.0 years; age range, 41–63 years) with a clinical diagnosis of breast cancer were enrolled in the study between May 2017 and July 2017. All patients underwent whole-body PET/CT scan with 16α-[^18^F]FES before surgery. Patients underwent the surgical operation within 1 month after the PET/CT scan, and definitive diagnosis was determined using postoperative pathohistology analysis. The study protocol was approved by the Sichuan Cancer Hospital Ethics Committee, and informed consent was obtained from all patients before the PET/CT scan.

All patients fasted 4 h before FES-PET. Approximately a dose of 148–222 MBq (4–6 mCi) 16α-[^18^F]FES was infused intravenously over 2 min. The whole-body scanning (2 min per table position) was performed 1 h after administration using a Siemens Biograph mCT-64 PET/CT scanner.

### Statistical Analysis

Means were compared using the two-tailed paired Student's *t* test. *P*-values <0.05 were considered significant. Data analyses were carried out using GraphPad Prism Software Version 5.0 (GraphPad Software Inc., CA, USA).

## Results

### [^18^F]Fluorination and Hydrolysis: K222 Catalyzed vs. TBA Catalyzed

**Figure 1:**
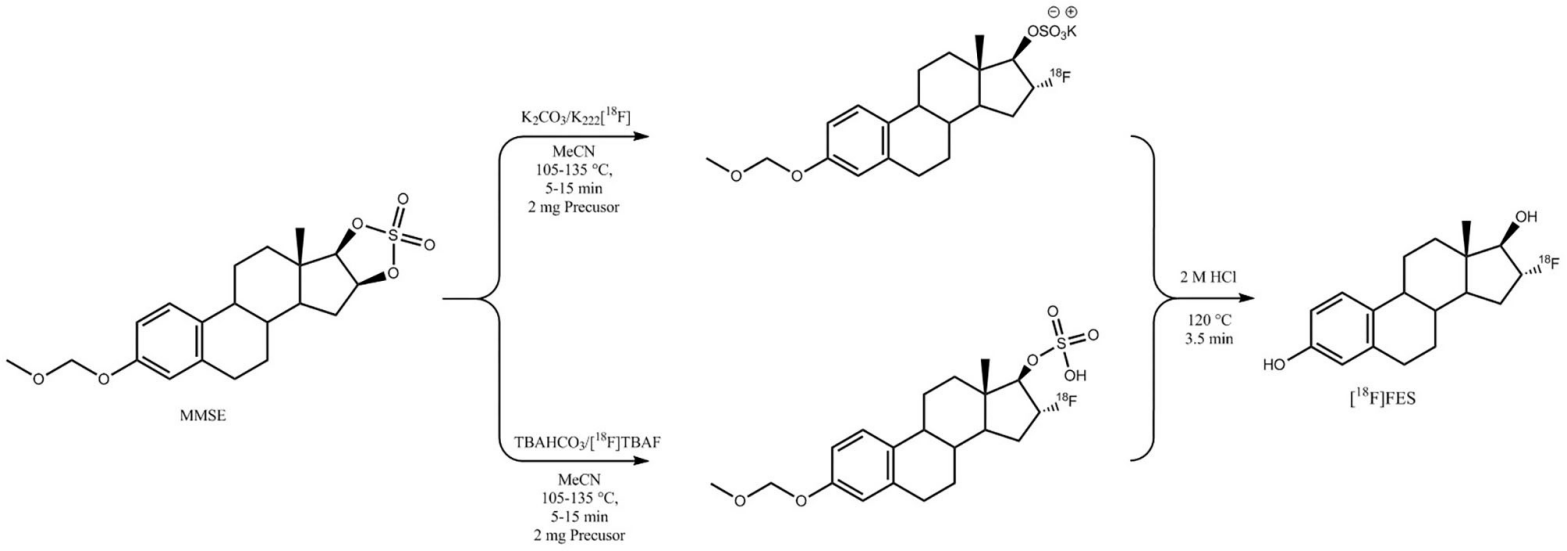
Scheme for the production of 16α-[^18^F]FES catalyzed by K222/K_2_CO_3_ or TBA·HCO_3_.

**Figure 2:**
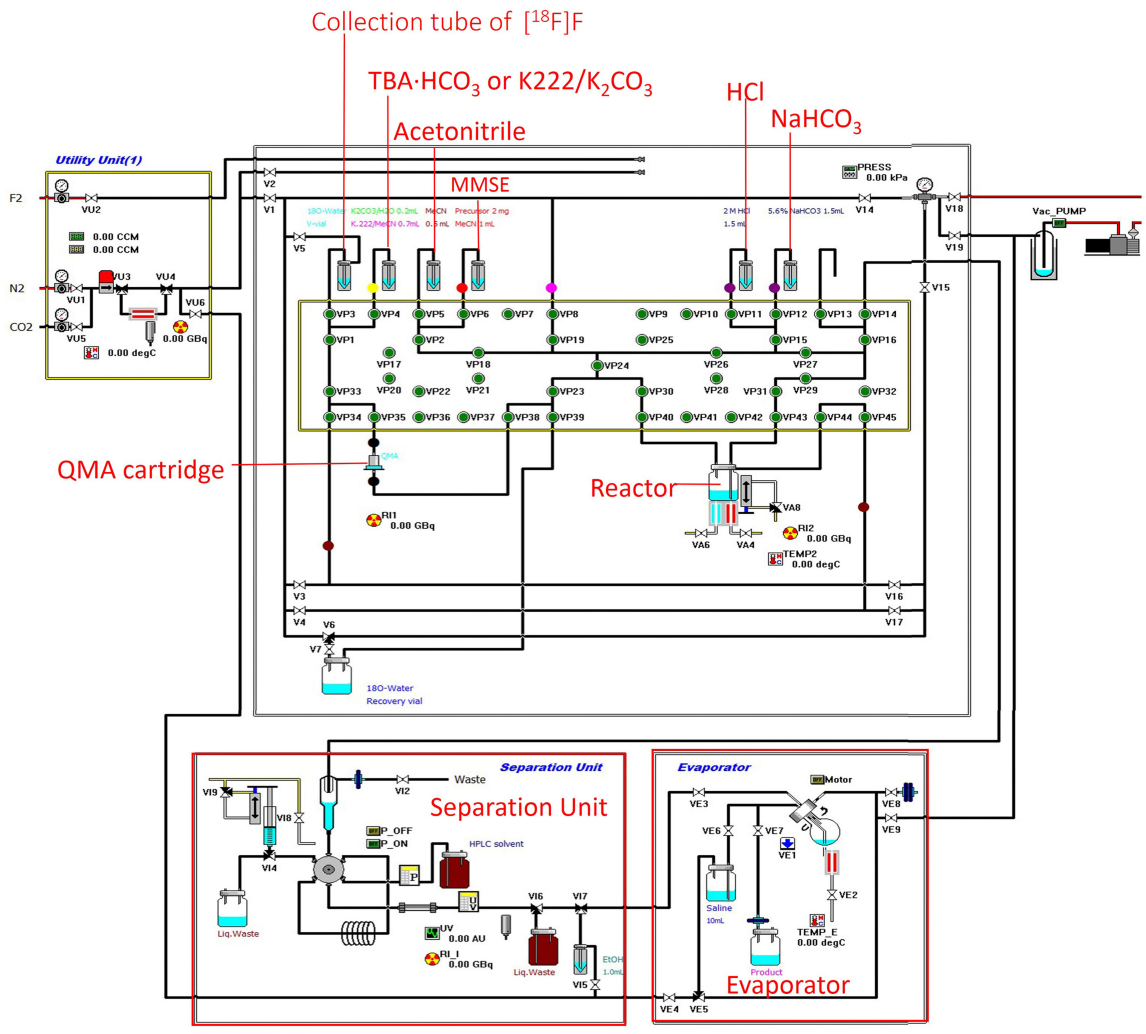
Modified hardware and reagent kits for 16α-[^18^F]FES based on the CFN-MPS-200 synthesis system.

**Figure 3:**
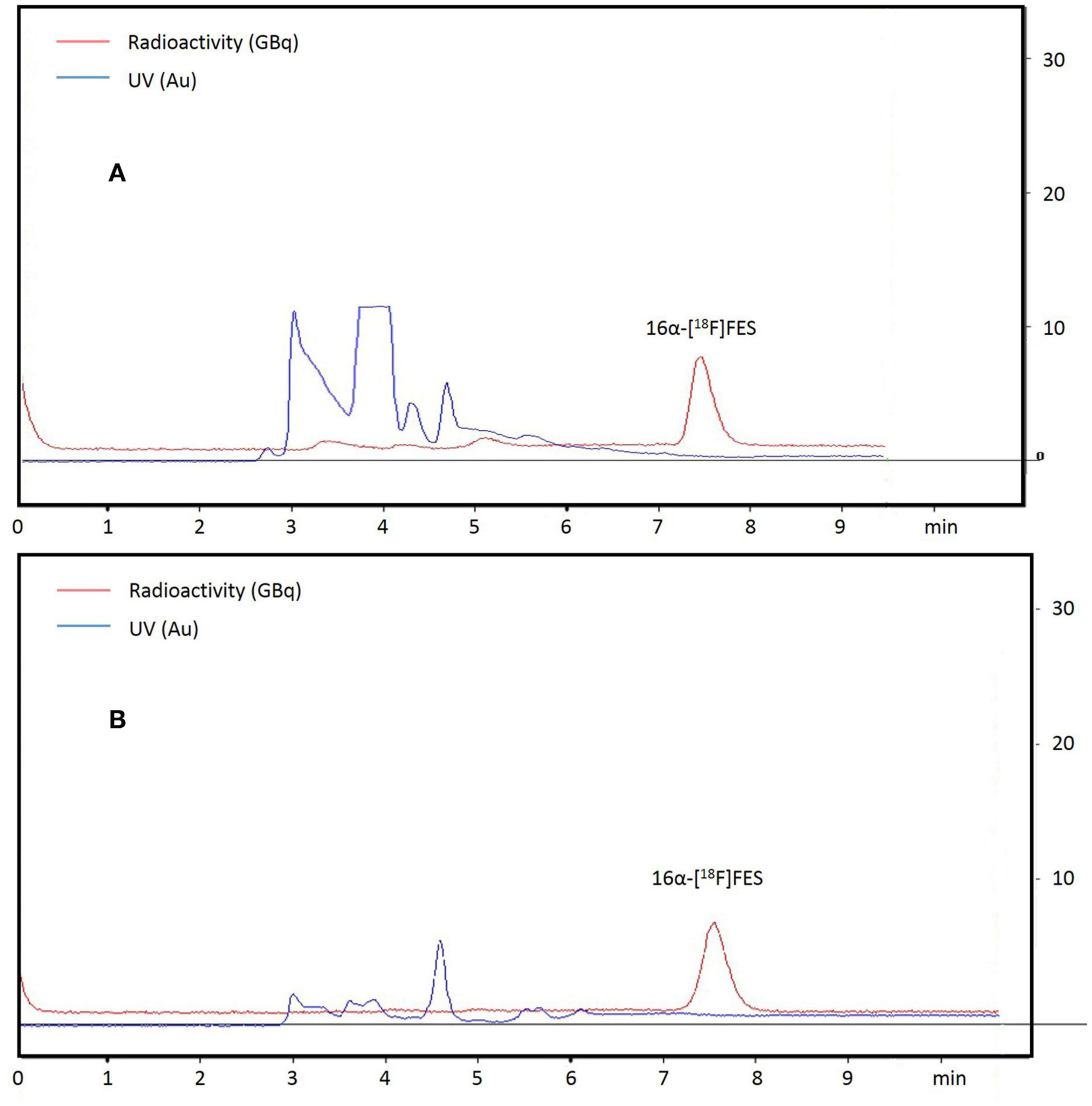
The semi-preparative chromatogram of crude 16α-[^18^F]FES, UV absorbance at 280 nm. **(A)** The chromatogram of the crude product (using K222/K_2_CO_3_). **(B)** The chromatogram of the crude product (using TBA·HCO_3_).

The procedures for the one-pot synthesis of 16α-[^18^F]FES were optimized and successfully performed (Figure 1). A total of 13 times synthesis were all successful, and the method was stable (Table 1). Hardware and reagent kits for 16α-[^18^F]FES using the CFN-MPS-200 synthesis system are shown in Figure 2. The commercially available precursor MMSE was used, and acetonitrile was chosen as the radiofluorination solvent instead of DMSO. Two different phase transfer catalysts (K222/K_2_CO_3_ and TBA·HCO_3_) were used, respectively, while the purification methods were the same (by semi-preparative HPLC purification). As purification results show in Figure 3A (K222/K_2_CO_3_ catalyzed) or Figure 3B (TBA·HCO_3_ catalyzed), only one radioactive peak was detected and collected, with a retention time of 7.5 min (using K222/K_2_CO_3_) or 7.6 min (using TBA·HCO_3_). After removing solvents by a rotary evaporator, the decay-corrected radiochemical yield of the final product 16α-[^18^F]FES was 48.7 ± 0.95% (using K222/K_2_CO_3_, *n* = 4) or 46.7 ± 0.77% (using TBA·HCO_3_, *n* = 4).

**Table 1:** Investigation of synthesis conditions.

**Entry**	**Precursor**	**Catalyst**	**Fluorination temperature (°C)**	**Fluorination time (min)**	**Radiochemical yield (%)**
1	2 mg MMSE/1 mL ACN	K222/K_2_CO_3_	115	15	46.4
2	2 mg MMSE/1 mL ACN	K222/K_2_CO_3_	115	15	47.8
3	2 mg MMSE/1 mL ACN	K222/K_2_CO_3_	115	15	49.8
4	2 mg MMSE/1 mL ACN	K222/K_2_CO_3_	115	15	50.6
5	2 mg MMSE/1 mL ACN	TBA·HCO_3_	135	10	45.6
6	2 mg MMSE/1 mL ACN	TBA·HCO_3_	135	10	47.0
7	2 mg MMSE/1 mL ACN	TBA·HCO_3_	135	10	45.5
8	2 mg MMSE/1 mL ACN	TBA·HCO_3_	135	10	48.8
9	2 mg MMSE/1 mL ACN	K222/K_2_CO_3_	115	5	36.7
10	2 mg MMSE/1 mL ACN	K222/K_2_CO_3_	115	10	41.3
11	2 mg MMSE/1 mL ACN	K222/K_2_CO_3_	105	15	37.5
12	2 mg MMSE/1 mL ACN	K222/K_2_CO_3_	125	15	50.4
13	2 mg MMSE/1 mL ACN	K222/K_2_CO_3_	135	15	51.6

*MMSE, 3-methoxymethyl-16β,17β-epiestriol-O-cylic sulfone; ACN, acetonitrile; K222, Kryptofix 222; TBA, tetrabutylammonium*.

### Quick Synthesis

16α-[^18^F]FES was synthesized rapidly in one pot with a high radiochemical yield and purity in this study. Overall, total K222-catalyzed synthesis time was 48 ± 2 min (*n* = 4) and total TBA-catalyzed synthesis time was 48 ± 3 min (*n* = 4); both included two azeotropic distillations of [^18^F]fluoride and acetonitrile, radiofluorination, hydrolysis, neutralization, HPLC purification, and desolventizing by rotary evaporator. In typical synthesis conditions, the six steps took the same time in both K222-catalyzed and TBA-catalyzed methods. Desolventizing by the rotary evaporator can reduce the solvent in the final formula to satisfy clinical use and takes about 10 min. If the synthesis of 16α-[^18^F]FES was aimed at research and development, omitting the desolventizing step could save 10 min and reduce product loss, which was stained with a rotary evaporator to obtain a high yield. Automated synthesis in commercially available synthesizer systems takes about 80 min, with a 10–20% uncorrected radiochemical yield (4–6).

### Rapid Quality Control of Radiochemical Purity and Chemical Purity

**Figure 4:**
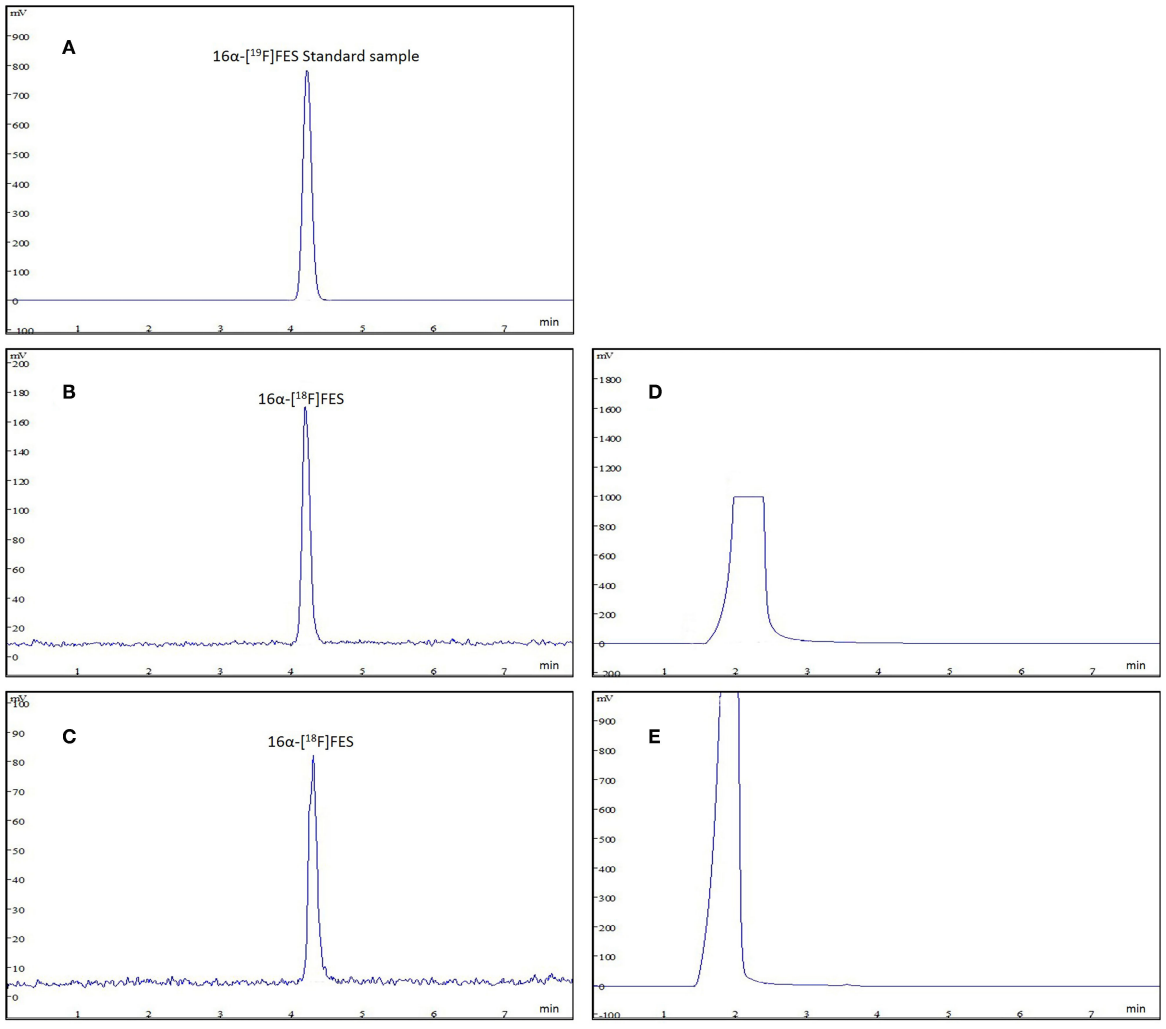
The analytic HPLC chromatogram of purified 16α-[^18^F]FES, UV absorbance at 280 nm. The UV absorbance and radioactive signals were both transformed into electrical signals. **(A)** UV absorbance of 16α-[^18^F]FES reference standard. **(B)** The chromatogram of product (using K222/K_2_CO_3_) measured by radioactive detector. **(C)** The chromatogram of product (using TBA·HCO_3_) measured by radioactive detector. **(D)** The chromatogram of product (using K222/K_2_CO_3_) measured by UV detector. **(E)** The chromatogram of product (using TBA·HCO_3_) measured by UV detector.

16α-[^18^F]FES is well known as an ideal PET tracer binding to ER. Radioactive impurities in 16α-[^18^F]FES product solution will influence the imaging for ER expression *in vivo*, and the high radiochemical purity of 16α-[^18^F]FES guarantees the accurate diagnosis. The final product was confirmed by comparing the product with the authentic reference standard. The analytical HPLC chromatograms (Figure 4) showed that the retention time was 4.2 min and only one radioactive peak was detected, which suggested that the radiochemical purity of product was almost 100%. In this study, 6α-[^18^F]FES with ideal radiochemical purity was produced and the high radiochemical purity might be due to the semi-preparative HPLC purification system instead of SPE cartridges.

The content of residual TBA shown was below 50 μg/ml according to the spot test. The value of the residual catalyst was below the limits stated in USP or Ph.Eur. (7, 8). Saline containing ethanol was used for the final product formula according to Mori (4). Analytical HPLC was used to determine the stability of the final product synthesized 8 h ago. The results showed that the long-term radiochemical purity of 16α-[^18^F]FES in saline containing ethanol at room temperature did not show any decomposition. There was no increase of chemical or radiochemical impurities. According to our study, purity, radiochemical yield, and stability between 16α-[^18^F]FES catalyzed by K222 and TBA showed no significant difference.

### 16α-[^18^F]FES PET/CT and ER Expressions

**Figure 5:**
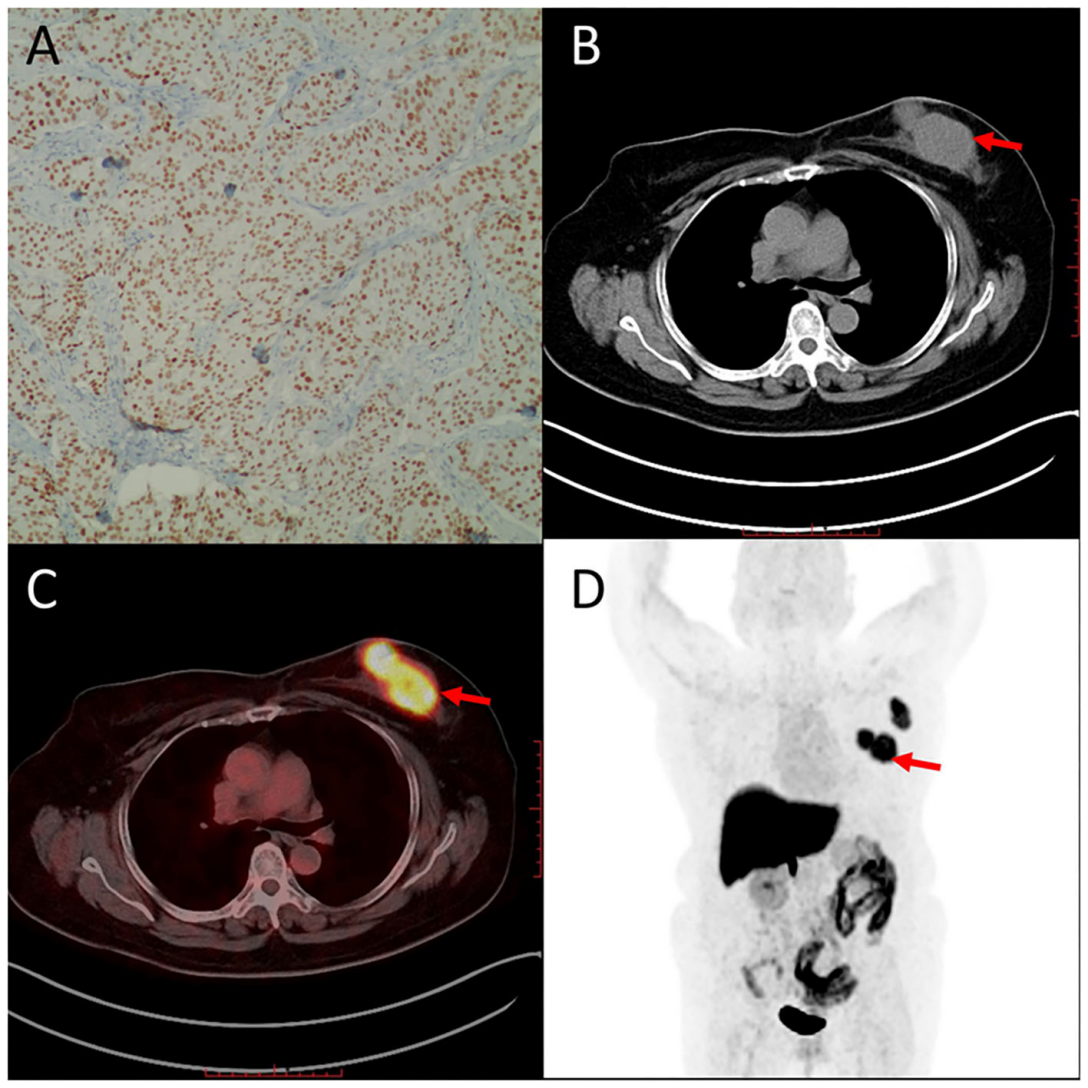
Representative case of ER-positive breast cancer on the upper outer quadrant of the left breast (55 years). Immunohistochemistry staining images are shown for ER **(A)**. 16α-[^18^F]FES PET/CT images **(B–D)** (red arrows) are shown. High accumulation of 16α-[^18^F]FES significantly corresponded with high expression of ER.

**Figure 6:**
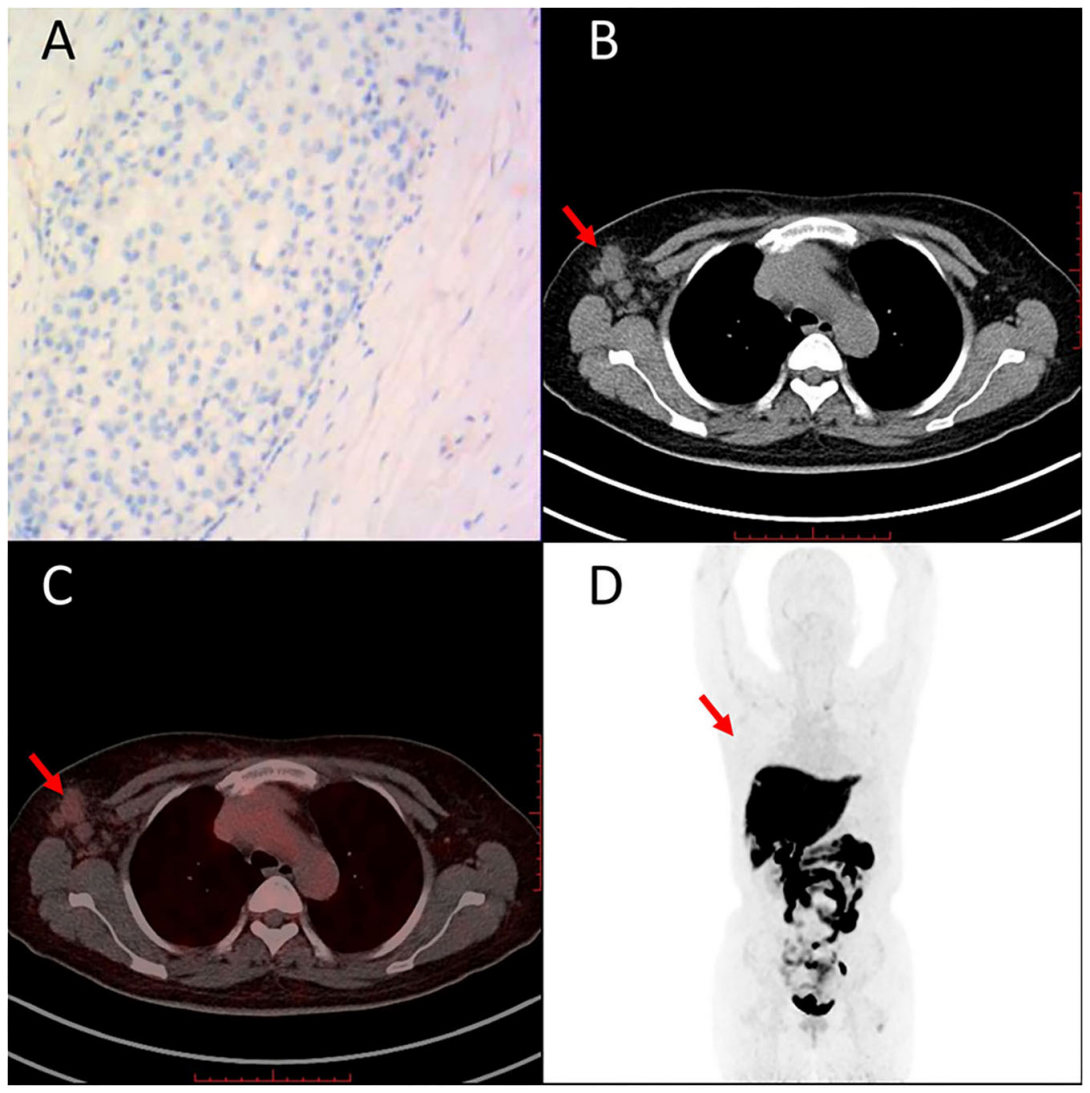
Representative case of ER-negative breast cancer on right axillary lymph node (49 years). Immunohistochemistry staining images are shown for ER **(A)**. 16α-[^18^F]FES PET/CT images **(B–D)** (red arrows) are shown. Low accumulation of 16α-[^18^F]FES significantly corresponded with low expression of ER.

Representative cases of clinically diagnosed breast cancer FES-PET are given in Figures 5, 6. Figure 5 shows a case with ER-positive breast cancer on the upper outer quadrant of the left breast. PET images showed high homogeneous 16α-[^18^F]FES accumulation, and immunohistochemical findings of this case are high expression of ER. The other case with ER-negative breast cancer on the right axillary lymph node (Figure 6) showed no uptake on PET images, and immunohistochemical findings of this case are no expression of ER.

## Discussion

Because the synthesis of PET tracer is the key link of whole PET examination processes, the successful synthesis of 16α-[^18^F]FES needs to be guaranteed. In this study, the one-pot synthesis of 16α-[^18^F]FES including six steps, azeotropic distillation, fluorination, acidic hydrolysis, NaHCO_3_ neutralization, semi-preparative HPLC purification, and solvent removal, was successfully performed. The residual water in the reactor often causes failure of fluorination in routine synthesis of 16α-[^18^F]FES. Therefore, two improvements were developed: one was that the reactor was dried up with an additional 0.5 ml of anhydrous acetonitrile before fluorination; the other was that the precursor MMSE would not be added in the reactor until it is confirmed, with the use of a camera, that the solution in the reactor was dried up and input into the computer. Because semi-preparative HPLC purified 16α-[^18^F]FES was stained with a rotary evaporator flask while removing the solvents, the radiochemical yield in this study was a little lower than that in previously reported studies (4, 9–11).

Water is not a suitable solvent for the synthesis of FES because fluorine has a high hydration energy. A polar aprotic solvent such as acetonitrile should be used in this S_N_2 nucleophilic substitution reaction.

The phase transfer catalyst plays an important role in the synthesis of various PET tracers. Many attempts have been made to develop nucleophilic substitution, which include ^18^F-CsF, ^18^F-Et_4_NF, and ^18^F-KHF. However, breakthrough of radiofluorination was not made until K222 was used as catalyst. Usually, the ^18^F^−^ washed out from the cyclotron target is accompanied by traces of metal ions from the surface of the target body. When passing through the light QMA anion exchange ion, the ^18^F^−^ is retained and the metal ions will be lost in the ^18^O-water. Hence, it is necessary to introduce a positively charged counter ion to restore the ^18^F^−^ reactivity before evaporation of residual ^18^O-enriched water (12). Several types of positively charged counter ions have been used, including potassium ion complexed by a large ring structure such as K222 and tetrabutylammonium salts (13). As Knott et al. (5) reported, using TBA·HCO_3_ in the synthesis of 16α-[^18^F]FES resulted in higher radioactive yield than using K222/K_2_CO_3_ when the fluorination condition was 8 min in 130°C. In this study, we further investigated whether K222/K_2_CO_3_ or TBA·HCO_3_ is the appropriate phase transfer catalyst for the automated synthesis of 16α-[^18^F]FES. The results showed that there was no significant difference in catalytic activity between K222/K_2_CO_3_ and TBA·HCO_3_ when the fluorination condition was 15 min in 135°C. It was a significant information for the routine synthesis of 16α-[^18^F]FES.

ERs are known as essential sex hormone receptors and are the predominant receptor in breast tissue. It is reported that 16α-[^18^F]FES PET can measure the *in vivo* ER expression of breast cancer noninvasively (14). In our study, 16α-[^18^F]FES uptake showed a significant correlation with ER expression in breast masses and axillary lymph node metastasis (Figures 5, 6). These results also reflect the successful synthesis of solvent-free 16α-[^18^F]FES in CFN-MPS-200 using module Kryptofix 222 or tetrabutylammonium bicarbonate.

## Conclusion

In this work, 13 times fully automatic synthesis of solvent-free 16α-[^18^F]FES had been successfully performed in the CFN-MPS-200 synthesis system through a six-step one-pot procedure with high radiochemical yield and radiochemical purity within a short time (<50 min). Solvent-free 16α-[^18^F]FES with high radiochemical purity was obtained with HPLC purification and desolventizing by a rotary evaporator and shows significant correlation with ER expression in patients. Moreover, two phase transfer catalysts, Kryptofix 222 and tetrabutylammonium bicarbonate, did not show significant difference in catalytic activity, radiochemical yield, radiochemical purity, and product stability in our study. In conclusion, we present a quick automatic method for synthesizing solvent-free 16α-[^18^F]FES that could be used in a commercial synthesis module with either Kryptofix 222 or tetrabutylammonium bicarbonate.

## Data Availability Statement

The original contributions presented in the study are included in the article/supplementary material, further inquiries can be directed to the corresponding author/s.

## Ethics Statement

The studies involving human participants were reviewed and approved by Sichuan Cancer Hospital Ethic Committee. The patients/participants provided their written informed consent to participate in this study.

## Author Contributions

XJ, YL, XW, TS, ZL, and ZC conceived and designed the study and helped to draft the manuscript. XL, YY, and GZ performed the data collection. YK and JS performed the statistical analysis. All authors read and critically revised the manuscript for intellectual content and approved the final manuscript.

## Conflict of Interest

The authors declare that the research was conducted in the absence of any commercial or financial relationships that could be construed as a potential conflict of interest.
